# Chemiluminescence Immunoassay Based Serological Immunoassays for Detection of SARS-CoV-2 Neutralizing Antibodies in COVID-19 Convalescent Patients and Vaccinated Population

**DOI:** 10.3390/v13081508

**Published:** 2021-07-30

**Authors:** Qiangling Yin, Yecheng Zhang, Lijun Lian, Yuanyuan Qu, Wei Wu, Zhen Chen, Rongjuan Pei, Tingyou Chen, Lina Sun, Chuan Li, Aqian Li, Jiandong Li, Dexin Li, Shiwen Wang, Wuxiang Guan, Mifang Liang

**Affiliations:** 1 State Key Laboratory for Molecular Virology and Genetic Engineering, National Institute for Viral Disease Control and Prevention, Chinese Center for Disease Control and Prevention, Beijing 102206, China; yinql18@163.com (Q.Y.); yyqu0114@163.com (Y.Q.); beilei0702@vip.sina.com (W.W.); linasun@yeah.net (L.S.); lic@ivdc.chinacdc.cn (C.L.); liaq@ivdc.chinacdc.cn (A.L.); ldong121@126.com (J.L.); lidx@chinacdc.cn (D.L.); 2 Center for Emerging Infectious Diseases, Wuhan Institute of Virology, Center for Biosafety Mega-Science, Chinese Academy of Sciences, Wuhan 430071, China; zyc414890146@foxmail.com (Y.Z.); chenzhen@wh.iov.cn (Z.C.); rongjuan_pei@wh.iov.cn (R.P.); 3 University of Chinese Academy of Sciences, Beijing 100049, China; 4 Innovita Biological Technology Co., Ltd., Beijing 100070, China; 13520201572@163.com (L.L.); chentingyou@innovita.com.cn (T.C.); 5 CDC-WIV Joint Research Center for Emerging Diseases and Biosafety, Wuhan 430071, China

**Keywords:** COVID-19, SARS-CoV-2, chemiluminescence immunoassay, neutralizing antibody, ACE2-RBD inhibition assay

## Abstract

The development of rapid serological detection methods re urgently needed for determination of neutralizing antibodies in sera. In this study, four rapid methods (ACE2-RBD inhibition assay, S1-IgG detection, RBD-IgG detection, and N-IgG detection) were established and evaluated based on chemiluminescence technology. For the first time, a broadly neutralizing antibody with high affinity was used as a standard for the quantitative detection of SARS-CoV-2 specific neutralizing antibodies in human sera. Sera from COVID-19 convalescent patients (N = 119), vaccinated donors (N = 86), and healthy donors (N = 299) confirmed by microneutralization test (MNT) were used to evaluate the above methods. The result showed that the ACE2-RBD inhibition assay calculated with either ACE2-RBD binding inhibition percentage rate or ACE2-RBD inhibiting antibody concentration were strongly correlated with MNT (r ≥ 0.78, *p* < 0.0001) and also highly consistent with MNT (Kappa Value ≥ 0.94, *p* < 0.01). There was also a strong correlation between the two evaluation indices (r ≥ 0.99, *p* < 0.0001). Meanwhile, S1-IgG and RBD-IgG quantitative detection were also significantly correlated with MNT (r ≥ 0.73, *p* < 0.0001), and both methods were highly correlated with each other (r ≥ 0.95, *p* < 0.0001). However, the concentration of N-IgG antibodies showed a lower correlation with the MNT results (r < 0.49, *p* < 0.0001). The diagnostic assays presented here could be used for the evaluation of SARS-CoV-2 vaccine immunization effect and serological diagnosis of COVID-19 patients, and could also have guiding significance for establishing other rapid serological methods to surrogate neutralization tests for SARS-CoV-2.

## 1. Introduction

The 2019 coronavirus disease (COVID-19) pandemic caused by SARS-CoV-2 has led to more than 170 million confirmed cases and more than 3.53 million deaths as of 31 May 2021 [1]. SARS-CoV-2 encodes four major structural proteins (spike (S), envelope (E), membrane (M), and nucleocapsid (N)) as well as nonstructural and accessory proteins [2]. The spike protein (S protein) on the surface of the virus envelope mediates virus adhesion and the invasion of host cells [3]. The S protein can be divided into two subunits: S1 and S2. Angiotensin converting enzyme 2 (ACE2) is the receptor for the virus which invades target cells [4]. S1 binds to ACE2 on the host cell through its receptor binding domain (RBD), initiates a conformational change of S2, causes virus-host cell membrane fusion, and promotes virus entry into the cell [5]. Almost all sera from COVID-19 convalescent patients show IgM or IgG positive to spike proteins [6,7,8,9]. The reported monoclonal neutralizing antibodies are specific to the S1 protein, targeting N-terminal domain (NTD) and RBD, and most of them bind to the RBD protein [10,11,12,13,14,15]. The neutralizing mechanism of NTD specific neutralizing antibodies is not clear. The RBD specific neutralizing antibody contains two epitopes: the ACE2 competitive epitope and the ACE2 non-competitive epitope [16]. Antibodies against the competitive epitope of ACE2 will inhibit the binding of RBD to ACE2, thereby blocking the entry of the virus into the host cell, inhibiting viral infection and exerting an antiviral effect [17]. Current public health measures did not prevent the virus from transmitting within countries and among countries. The development and usage of vaccines represents the only effective way to control the global epidemic [18].

There are 284 ongoing SARS-CoV-2 vaccine research projects around the world based on the full-virus inactivated vaccine, subunit vaccine, mRNA vaccine, and viral vector vaccine. Among them, 101 projects have entered the clinical trial stage [19]. More than 3.58 billion doses of vaccines have been administered globally, and 1.4 billion doses of vaccines have been administered in China [20]. After vaccination, the evaluation of population immunization effect is very important for epidemic prevention and control, and neutralizing antibody titer in sera is an important indicator of immune effect. Neutralization assay, a conventional method to detect titer of sera neutralizing antibodies, involves the use of live viruses and needs to be carried out in a biological safety level 3 (BSL-3) Laboratory, which has several disadvantages such as having bio-safety risks, high professional ability requirements for operators, and being time consuming, costly, and not conducive to high throughput detection [21,22]. Therefore, it is necessary to develop a cost efficient, fast and large-scale alternative neutralizing antibody detection method.

Chemiluminescence Assay (CLIA) is an assay that combines the chemiluminescence technique with immunochemical reactions [23,24,25], which is reproducibility, cost effectiveness, fast and precise measurement of the IgG and IgM antibody levels [26]. In this study, the ACE2-RBD Inhibition Assay method, anti-SARS-CoV-2 S1 protein immunoglobulin G (S1-IgG) detection method, anti-SARS-CoV-2 RBD protein immunoglobulin G (RBD-IgG) detection method, and anti-SARS-CoV-2 N protein immunoglobulin G (N-IgG) detection method were constructed based on chemiluminescence technology. These methods was evaluated with 119 sera from COVID-19 convalescent patients and 86 sera from SARS-CoV-2 vaccinated donors. The correlation between the four methods and neutralization assay was analyzed to evaluate the ability of the four detection methods to detect neutralizing antibodies in the sera of COVID-19 convalescent patients and the vaccinated donors.

## 2. Materials and Methods

### 2.1. Sera

COVID-19 patients sera used in this study were randomly collected from COVID-19 patients in convalescent phase with real time PCR confirmed SARS-CoV-2 infection (N = 119). All of the vaccinated sera used in this study were randomly collected from volunteers two weeks after receiving the second dose inactivated vaccine (Sinopharm or Sinovac) (N = 86). And negative sera were obtained from healthy donors prior to the COVID-19 pandemic between 2018 and 2019 (N = 299). All sera above were used in microneutralization assay, ACE2-RBD inhibition assay and Anti-SARS-CoV-2-IgG detection assay (S1 protein, RBD protein and N protein). All participants in this study gave written informed consent before participation in this study.

### 2.2. Proteins

8× His tag labeled human ACE2 (hACE2-his), human IgG1 Fc tag labeled human ACE2 (hACE2-Fc), 8×His tag labeled SARS-CoV-2 RBD (RBD-his), human IgG1 Fc tag labeled RBD (RBD-Fc) and SARS-CoV-2 S1 used in this study were purchased from Jiangsu East-mab Biomedical Technological Co., Ltd. (Jiangsu East-mab, Jiangsu, China). The protein sequence of human ACE2 was from the NCBI GenBank database (AA 18–740, GenBank accession number NM_021804. The protein sequences of S1 (AA 16–685) and RBD (AA 319–541) were from spike protein of SARS-CoV-2 reference strain (Genbank: MN996528.1). The nucleocapsid phosphoprotein (Full length, GenBank accession number QQM17835.1) was expressed and purified in our laboratory as described before [27].

### 2.3. Human Monoclonal Antibodies

The human monoclonal antibodies targeting RBD (F61, F163, H121, C25) and N protein (N44) of SARS-CoV-2 were screened by phage display technology [28]. Briefly, the variable regions of antibodies were amplified from the lymphocytes isolated from COVID-19 convalescent donors and then cloned into the pComb 3H vector. The vectors were then transformed into *E. coli* to construct human antibody phage libraries. The final yielded antibody libraries were panned and screened with purified SARS-CoV-2 S1 protein, RBD protein and N protein. Eventually, four high affinity RBD specific monoclonal antibodies F61, F163, H121, C25 (KD range from 2.83 × 10^−12^ M to 4.10 × 10^−11^ M) and one high affinity N specific monoclonal antibodies N44 (KD = 2.83 × 10^−10^ M) were picked and used as candidate standards in this study [28].

### 2.4. ACE2-RBD Inhibition Assay

**Figure 1:**
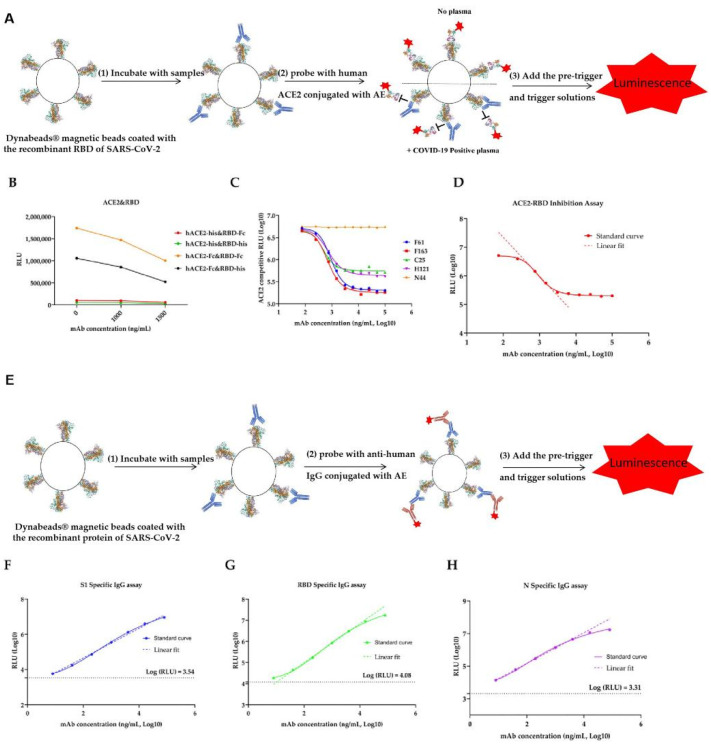
The methods of ACE2-RBD-inhibiting Ab and SARS-CoV-2 specific IgG test construction. (**A**) Graphic representation of ACE2-RBD inhibition assay. (**B**) Optimization of the antigen using in ACE2-RBD inhibition assay. (**C**) Selection of antibody standards from F61, F163, H121, C25, N44 for ACE2-RBD inhibition assay. (**D**) Standard curve for ACE2-RBD-inhibiting mAb F163. Linear range of standard curve were determined using linear regression curves: Y = −1.364X + 10.08, R2 = 1.0000. (**E**) Graphic representation of anti-SARS-CoV-2 IgG detection using S1 segment of spike protein, receptor binding domain (RBD) and nucleoprotein. (**F**–**H**) Standard curve for S1-IgG, RBD-IgG, and N-IgG, mAb F163 was used as standards for S1-IgG and RBD-IgG detection, mAb N44 was used as standards for N-IgG. Linear range of standard curve were determined using linear regression curves: Y = 0.8393 + 2.983, R2 = 0.9939 for S1-IgG; Y = 0.9263X + 3.139, R2 = 0.9998 for RBD-IgG; Y = 0.9423X + 3.268, R2 = 0.9998 for N-IgG, respectively. The background RLU of the anti-human IgG-AE alone on the S1, RBD and N labeled beads were displayed as a dotted line parallel to the x axis.

Dynabeads^®^ magnetic beads (purchased from Thermo Scientific, Waltham, MA, USA) were coated with the recombinant RBD of SARS-CoV-2 through cross linking by N-ethyl-N^®^ (3-dimethylamino-propyl) carbodiimide (purchased from Thermo Scientific). Human ACE2-hFc and human ACE2-8×his were conjugated with acridinium ester (AEs were purchased from Xiamen Heliosense Company, Xiamen, Fujian, China), and the conjugated ACE2 were then purified by column filtration on a GE PD-10 column. The calibrators were made from monoclonal antibodies targeting SARS-CoV-2 RBD with designated concentrations (ng/mL). The pre-trigger and trigger solutions are composed of sodium hydroxide and hydrogen peroxide. All measuring procedures were established based on the ACL2800 automatic chemiluminescent analyzer provided by Nanjing DGNS Biotech Co., Ltd. (Nanjing, Jiangsu, China). The correlation between the chemiluminescent signal measured as relative light unit (RLU) and the concentration of SARS-CoV-2 IgG is shown in the Standard curves. In Figure 1, A is reported the graphic representation of ACE-RBD inhibition assay (Figure 1A).

The SARS-CoV-2 ACE2-RBD-inhibiting assays are two step immunoassays for the Quantitative detection of ACE2-RBD inhibiting antibodies in human sera using direct chemiluminometric microparticle technology. All samples were 1:20 diluted before detection. In the first step, a 20 μL sample and 20 μL of recombinant SARS-CoV-2 RBD-coated Dynabeads^®^ magnetic beads (Thermo Scientific, Waltham, MA, USA) were combined. After 20 min incubation in 37 °C, ACE2-RBD-inhibiting antibodies present in the sample bound to the SARS-CoV-2 RBD-coated magnetic beads. Then, 40 μL of AE-labeled ACE2 conjugate was added, and could not form an ACE2-RBD reaction complex in the second step if the antibody present in the sample were targeting ACE2-RBD binding domain. Following a wash cycle, pre-trigger and trigger solutions were added to the reaction mixture. The resulting chemiluminescent reaction was measured as RLU. An inverse relationship exists between the amount of SARS-CoV-2 ACE2-RBD-inhibiting antibodies in the sample and the RLU detected by the optical system of the automatic chemiluminescent analyzer. The concentration of SARS-CoV-2 ACE2 competitive antibodies in the sample is determined by comparing the RLU of a sample to the RLU determined from Standard curves (Figure 1D).

### 2.5. S1, RBD and N Specific IgG Antibody Detection

Dynabeads^®^ magnetic beads were coated with the recombinant proteins of SARS-CoV-2 through cross-linking by N-ethyl-N^®^ carbodiimide. Human monoclonal anti-human IgG (purchased from Fapon Biotech, Guangdong, Dongguan, China) were conjugated with acridinium ester (AE), and the conjugated antibodies were then purified by column filtration on a GE PD-10 column. The calibrators were made from monoclonal antibodies targeting SARS-CoV-2 recombinant proteins with designated concentrations (ng/mL). The pre-trigger and trigger solutions were composed of sodium hydroxide and hydrogen peroxide. All measuring procedures were established based on the ACL2800 automatic chemiluminescent analyzer provided by Nanjing DGNS Biotech Co., Ltd. (Nanjing, Jiangsu, China). The correlation between the chemiluminescent signal measured as relative light unit (RLU), and the concentration of SARS-CoV-2 IgG is shown in the standard curves (Figure 1E).

The SARS-CoV-2 IgG assays are two step immunoassays for the quantitative detection of SARS-CoV-2 IgG antibodies in human Sera using direct chemiluminometric microparticle technology. In the first step, a 20 μL sample and 20 μL of recombinant SARS-CoV-2 antigen coated Dynabeads^®^ magnetic beads were combined. After 6 min incubation in 37 °C, SARS-CoV-2 specific IgG antibodies present in the sample bound to the SARS-CoV-2 antigens-coated magnetic beads. After washing, 40 μL AE-labeled anti-human IgG conjugate was added to form a reaction complex in the second step. Following another wash cycle, pre-trigger and trigger solutions were added to the reaction mixture. The resulting chemiluminescent reaction was measured as RLU. A positive correlation exists between the amount of SARS-CoV-2 specific IgG antibodies in the sample and the RLU detected by the optical system of the automatic chemiluminescent analyzer. The concentration of SARS-CoV-2 IgG in the sample was determined by comparing the RLU of a sample to the RLU determined from the standard curves (Figure 1F–H).

### 2.6. Microneutralization Test (MNT)

The microneutralization test is considered to be the gold standard for characterizing neutralizing antibodies to most viruses, including SARS-CoV-2. All sera samples were tested for neutralization against a SARS-CoV-2 reference strain (Genbank: MN996528.1) in the bio-safety Level 3 (BSL-3) laboratory. The assay was performed as described by Manenti et al. with a few modifications [29]. Briefly, sera samples were heat inactivated for 30 min at 56 °C, two-fold serially (1:5 to 1:4860) diluted heat inactivated sera samples (50 μL) in minimal essential medium (Gibco, Thermo Fisher Scientific, Waltham, MA, USA) supplemented with two percent fetal bovine sera (Gibco, Thermo Fisher Scientific, Waltham, MA, USA) were prepared (four replicates per dilution). In the next step, 50 μL of virus suspension of 100 tissue culture infective dose of previously titrated virus stock was added to each well of a 96 well plate (Greiner bio-one GmbH, Frickenhausen, Germany) and incubated at 37 °C for one hour. 100 μL of Vero E6 cells (1 × 10^5^ cells/mL) was then added to the 96 well plates and incubated at 37 °C with 5% CO_2_. After incubation for 72 h, cytopathic effect (CPE) was observed under a light microscope (Nikon, ×100, Tokyo, Japan). The number of positive holes in each row was counted and the Sera neutralization titer was calculated using the Reed–Muench method. A cutoff of neutralization titre >1:10 was considered as positive.

### 2.7. Statistical Analyses

Comparisons between two groups were made using two tailed t tests. Linear data were analyzed using an unpaired t test with Welch’s correction (for 2 groups) and were correlated using nonparametric tests (Kruskall–Wallis test and spearman’s correlation) A total of 504 sera samples, including the 205 positive and 299 negative samples as confirmed by the MNT, were tested by CLIA. The end point cutoff was determined by the analysis of a receiver operating characteristic (ROC) curve based on positive divided by negative (P/N) values. The panels of sera samples described above were selected to validate cross reactivity. Standard curve was determined using simple linear regressions or four-parameter logistic regressions with default settings, and r^2^ values were reported. Kappa values were calculated. The kappa statistic (k) was also used as an indicator of the diagnostic agreement between MNT and the four rapid diagnostic methods.

## 3. Results

### 3.1. Establishment and Optimization of the Detection Method

Given the current needs for SARS-CoV-2 serological testing, we developed a serological assay using chemiluminometric microparticle technology. To optimize the ACE2-RBD binding inhibition assay, hACE2 and RBD labeled by different tags were coupled to detected ACE2-RBD inhibiting antibody. When hACE2-Fc and RBD-Fc were paired, the RLU decreased significantly with the increase of antibody concentration in sera (Figure 1B). The results showed that F61 and F163 were competitive most obviously and could be used as standards for ACE2-RBD inhibition test (Figure 1C). F163 was used as standard substance to establish a standard curve of ACE2-RBD inhibition assay. S1-IgG detection, RBD-IgG detection, and N44 was used as standard substance to establish an N-IgG detection standard curve. The linear range of ACE2-RBD inhibition assay was 463–1818 ng/mL (Figure 1D), and the linear range of S1-IgG, RBD-IgG and N-IgG was 8–80,000 ng/mL, 30–5891 ng/mL and 8–4000 ng/mL, respectively (Figure 1F–H).

### 3.2. Detection Result of Sera from COVID-19 Convalescent Patients, Vaccinated Donors and Healthy Donor

Sera samples from COVID-19 patients in the convalescent phase (N = 119), vaccinated donors (N = 86), and healthy donors (N = 299) were included in this study. We analyzed the neutralization antibody titer in sera used in this study by a microneutralization test (MNT). The distribution of neutralization antibody titer exhibited in Table S1 was only applicable to sera samples involved in this study. The neutralization antibody titer in sera from COVID-19 convalescent patients ranged from 1:5 to 1:464. About 94.12% (112/119) of them were considered as positive with a neutralization antibody titer more than 1:10. To be specific, 5.88% (7/119) of them had neutralization antibody titer less than 1:10, 42.86% (51/119) of them were between 1:10 and 1:160, 32.77% (39/119) were between 1:160 and 1:500, and 18.49% were over 1:500. All of the vaccinated sera (N = 86) were positive with the neutralization antibody titer ranging from 1:24 to 1:768. Over half of them (58.14%) showed a neutralization antibody titer between 1:10 and 1:160, while 30.23% of them were between 1:160 and 1:500. All of the sera from healthy donors were negative (data not show).

**Figure 2:**
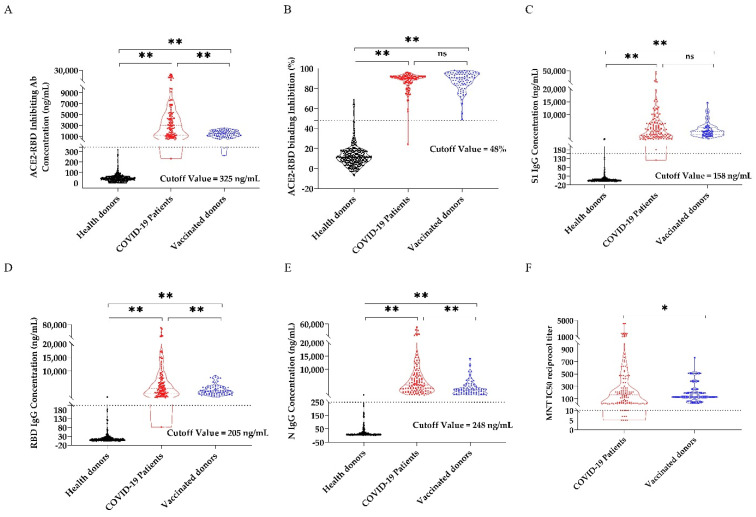
ACE2-RBD-inhibiting antibody, SARS-CoV-2 specific IgG and microneutralization test results of 119 convalescent samples, 86 vaccinated samples and 299 health control samples. (**A**) ACE2-RBD-inhibiting antibody concentration. (**B**) ACE2-RBD binding inhibtion. (**C**) S1-IgG. (**D**) RBD-IgG. (**E**) N-IgG concentrations. (**F**) Neutralizing antibody titer of 119 convalescent samples, 86 vaccinated samples, and 299 health control samples were measured using CLIA and MNT. The 119 convalescent samples results are shown as red dots, the 86 vaccinated samples results are shown as blue dots, and the 299 health control samples results are shown as black dots. Dashed lines represent the cutoff for positive sample designation, determined by ROC curve. Results between each paired groups were analysis by t test, and means significant differences between two groups were shown as ** (*p* < 0.01) and * (*p* < 0.05). No significant difference was shown as ns, *p* > 0.05.

Then, the methods established and optimized above were used to detect the SARS-CoV-2 specific antibody in sera samples verified by MNT. Antibody concentration was calculated based on standard curve established above (Figure 1D,F–H). At the same time, the percentage of ACE2-RBD binding inhibition was calculated (Figure 2B). The cutoff value was determined by an ROC curve (Supplementary Figure S1 and Table S2).

In the ACE2-RBD binding inhibition assay, ACE2-RBD inhibiting antibody concentration were ranging from 232 ng/mL to 22,226 ng/mL in convalescent sera and 263 ng/mL–2523 ng/mL in vaccinated sera. 99.15% (118/119) of convalescent sera and 98.83% (85/86) of vaccinated sera showed neutralization antibody positive with ACE2-RBD inhibiting antibody concentration more than 325 ng/mL. Most of sera from healthy donors (97.99%) was detected lower than the cut off value (Figure 2A). When the inhibition rate was used as the evaluation index, the result was similar. 99.15% (118/119) of convalescent sera and 100% (86/86) of vaccinated sera showed a neutralization antibody positive with ACE2-RBD inhibition rate higher than the cutoff value of 48% (Figure 2B).

In the S1 specific IgG antibody detection assay, the concentration of S1-IgG antibody was ranging from 121 ng/mL to 47,130 ng/mL in convalescent sera and 561 ng/mL to 14,714 ng/mL in vaccinated sera. 99.15% (118/119) of convalescent sera and 100% (86/86) of vaccinated sera showed neutralization antibody positive with S1-IgG antibody concentration higher than the cutoff value (158 ng/mL). Only 1.6% (5/299) of sera from healthy donors was detected positive (Figure 2C). Similarly, in the RBD specific IgG antibody detection assay, 99.15% (118/119) of convalescent sera and 100% (86/86) of vaccinated sera showed positive with RBD-IgG antibody higher than 205 ng/mL. 97.99% (293/299) of sera from healthy donor was detected negative with the concentration of RBD-IgG antibody lower than 205.4 ng/mL (Figure 2D). For N-IgG detection assay, all samples from COVID-19 convalescent patients and vaccinated people were above the cutoff value (248 ng/mL) (Figure 2E).

### 3.3. Correlation Analysis among ACE2-RBD Inhibition Assay, S1-IgG Assay, RBD-IgG Assay and N-IgG Assay

**Figure 3:**
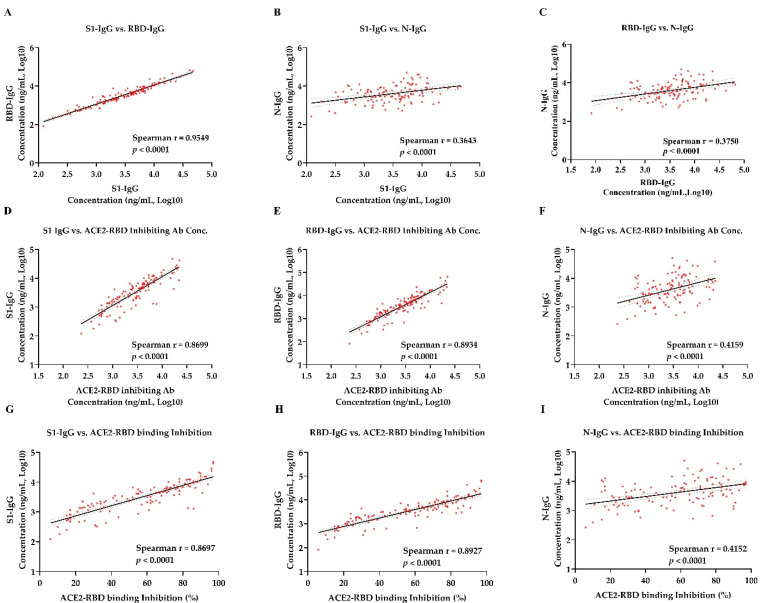
Correlation of ACE2-RBD inhibition assay and SARS-CoV-2 specific IgG concentration tests in 119 convalescent samples. (**A**) Correlation of S1-IgG and RBD-IgG. (**B**) Correlation of S1-IgG and N-IgG. (**C**) Correlation of RBD-IgG and N-IgG. (**D**–**F**) Correlation of ACE2-RBD inhibiting antibody concentration and S1-IgG, RBD-IgG, N-IgG. (**G**–**I**) Correlation of ACE2-RBD binding inhibition were computed by nonparametric spearman correlation. Spearman r > 0.7 and *p* value < 0.05 indicate a strong correlation, Spearman r < 0.7 and *p* value < 0.05 indicate a weak correlation, while *p* value > 0.05 indicate no correlation. The solid line represents a linear regression, with 95% confidence intervals represented by dotted lines.

**Figure 4:**
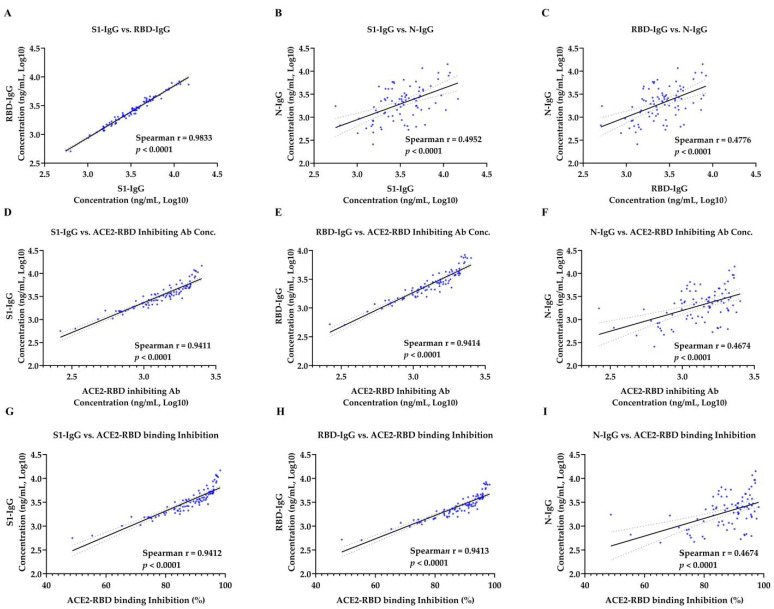
Correlation of ACE2-RBD inhibition assay and SARS-CoV-2 specific IgG concentration tests in 86 vaccinated samples. (**A**) Correlation of S1-IgG and RBD-IgG. (**B**) Correlation of S1-IgG and N-IgG. (**C**) Correlation of RBD-IgG and N-IgG. (**D**–**F**) Correlation of ACE2-RBD inhibiting antibody concentration and S1-IgG, RBD-IgG, and N-IgG. (**G**–**I**) Correlation of ACE2-RBD binding inhibition were computed by nonparametric spearman correlation. Spearman r > 0.7 and *p* value < 0.05 indicate a strong correlation, Spearman r < 0.7 and *p* value < 0.05 indicate a weak correlation, while *p* value > 0.05 indicate no correlation. The solid line represents a linear regression, with 95% confidence intervals represented by dotted lines.

Spearman correlation was used to analyze the correlation between antibody concentrations in sera samples from patients in the convalesce period of COVID-19 (Table 1) and vaccinated donors (Table 2). There was a strong correlation between the concentration of S1-IgG antibody and RBD-IgG antibody in COVID-19 convalescent sera (r = 0.955, *p* < 0.0001) and vaccinated sera (r = 0.983, *p* < 0.0001) (Figure 3A and Figure 4A). However, a moderate correlation was observed between S1-IgG assay and N-IgG assay (r = 0.3643 for convalescent sera and r = 0.495 for vaccinated sera, *p* < 0.0001) (Figure 3B and Figure 4B). So as to the correlation between the concentration of RBD-IgG antibodies and N-IgG antibodies, with r = 0.375 for convalescent sera (Figure 3C) and r = 0.478 for vaccinated sera (Figure 4C), respectively. Strong correlations were observed between the ACE2-RBD inhibition assay and anti–SARS-CoV-2 S protein IgG assay. ACE2-RBD binding inhibition rate were strongly correlated with the concentration of S1-IgG antibody, with the r = 0.8697 for convalescent sera (Figure 3D) and r = 0.9412 for vaccinated sera (Figure 4D), respectively. There was significant correlation between the concentration of the ACE2-RBD inhibitory antibody and S1-IgG antibody, with the spearman coefficient of 0.8699 for the convalescent sera (Figure 3G) and 0.9411 for the vaccinated sera (Figure 4G), respectively. Similarly, ACE2-RBD binding inhibition rate were strongly correlated with the concentration of RBD-IgG antibody. The spearman coefficient for COVID-19 convalescent sera and vaccinated sera were 0.8927 (Figure 3E) and 0.9413 (Figure 4E), respectively. The spearman coefficient between ACE2-RBD inhibiting antibody concentration and RBD-IgG antibody concentration were 0.8934 for convalescent sera (Figure 3H) and 0.9414 for vaccinated sera (Figure 4H). However, there was a moderate correlation between ACE2-RBD-inhibition assay and N-IgG antibody concentration. The spearman coefficient between the ACE2-RBD binding inhibition percentage rate and N-IgG antibody concentration for COVID-19 convalescent sera and vaccinated sera were 0.4152 (Figure 3F) and 0.4674 (Figure 4F). The spearman coefficient between ACE2-RBD inhibiting antibody concentration and N-IgG antibody concentration were 0.4159 for convalescent sera (Figure 3I) and 0.4674 for vaccinated sera (Figure 4I).

### 3.4. Correlation Analysis of Micro-Neutralization Test with ACE2-RBD Inhibition Assay, S1-IgG Assay, RBD-IgG Assay and N-IgG Assay

**Figure 5:**
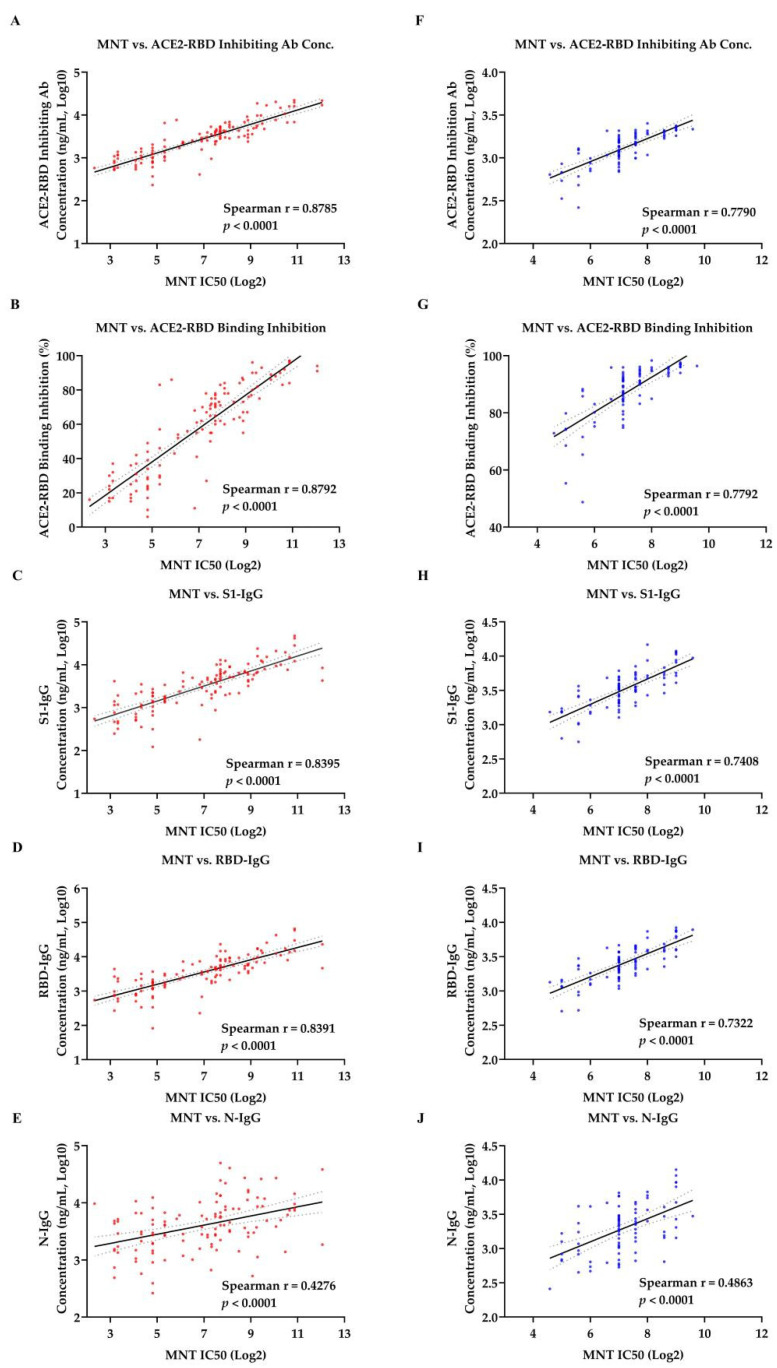
Correlation among microneutralization test (MNT), ACE2-RBD inhibition assay or SARS-CoV-2 specific IgG tests in samples. Correlation of 119 convalescent samples (**A**–**E**) and 86 vaccinated samples (**F**–**J**) test results were computed by nonparametric spearman correlation. (**A**,**F**) Correlation of MNT and ACE2-RBD inhibiting Antibody concentration. (**B**,**G**) Correlation of MNT and ACE2-RBD binding inhibition. (**C**,**H**) Correlation of MNT and S1-IgG. (**D**,**I**) Correlation of MNT and RBD-IgG. (**E**,**J**) Correlation of MNT and N-IgG. Spearman r > 0.7 and *p* value < 0.05 indicate a strong correlation, Spearman r < 0.7 and *p* value < 0.05 indicate a weak correlation, while *p* value > 0.05 indicate no correlation. The solid line represent a linear regression, with 95% confidence intervals represented by dotted lines.

Spearman correlation was used to analyze the correlation between SARS-CoV-2 specific antibodies concentrations and neutralizing antibody titer in sera samples from patients in the convalesce period of COVID-19 (Table 1) and vaccinated donors (Table 2). In the ACE2-RBD-inhibition assay, both ACE2-RBD-inhibiting antibody concentration (r = 0.8785 for convalescent sera and r = 0.7790 for vaccinated sera, *p* < 0.0001) and ACE2-RBD binding inhibition rate (r = 0.8792 for convalescent sera and r = 0.7792 for vaccinated sera, *p* < 0.0001) showed strong correlations with results of MNT (Figure 5A,B,F,G). Similarly, strong correlations were also observed between the results of MNT and anti–SARS-CoV-2 S protein IgG. To be specific, neutralization titer were strongly correlated with concentrations of theS1-IgG antibody and RBD-IgG antibody for convalescent sera (r = 0.8391 and 0.8395, respectively, *p* < 0.0001) and for vaccinated sera (r = 0.7322 and 0.7408, respectively, *p* < 0.0001) (Figure 5C,D,H,I). However, weak correlations between the neutralization titer and concentration of N-IgG antibody were observed both in convalescent sera (r = 0.4276, *p* < 0.0001) and vaccinated sera (r = 0.4863, *p* < 0.0001) (Figure 5E,G).

### 3.5. Consistency Analysis of Chemiluminescence Immunoassays and Microneutralization Test Results

We then analyzed the consistency between four rapid serological detection methods based on chemiluminometric microparticle technology developed above and microneutralization test (MNT). These four methods showed high consistency with MNT (kappa value = 0.9419, 0.9419, 0.9461, 0.9585 and 0.9378, respectively, *p* < 0.01). All of these methods had high sensitivity and specificity. To be specific, the sensitivity and specificity of ACE2-RBD binding inhibition rate were 99.49% and 95.75%, respectively. Similarly, the sensitivity and specificity of ACE2-RBD inhibitory antibody concentration was 99.49% and 95.75%. However, the sensitivity of S1-IgG assay, RBD-IgG assay, N-IgG assay were 99.49%, 99.49%, and 100%, respectively. The specificity of these three assays were 96.08%, 97.06%, and 95.10% respectively (Table 3 and Table S3).

Rapid global transmission of SARS-CoV-2 poses a serious threat to human health [1]. With large-scale vaccination, it is necessary to conduct massive population serological detections to determine the sera positive rate of the SARS-CoV-2 antibody, so as to evaluate the population immunization. The standard method of neutralizing antibody detection is a live virus neutralization test, which needs to be carried out in the bio-safety level-3 (BSL-3) laboratory. At the same time, the operator needs professional bio-safety training and experimental operation skills training. The detection is time consuming, costly, and does not involve high-throughput detection [21,22]. Therefore, it is necessary to establish an alternative detection method for neutralizing antibodies. It is reported that SARS-CoV-2 RBD is immunodominant and accounts for 90% of sera neutralizing activity [16]. Based on this feature, this study established ACE2-RBD inhibition assay, S1-IgG, RBD-IgG, and N-IgG detection methods. The detection method was evaluated with 119 sera from convalescent patients and 86 sera after immunization with SARS-CoV-2 vaccine. The correlation between SARS-CoV-2 specific antibodies was analyzed. It was found that there is a strong correlation between S1-IgG and RBD-IgG, while N-IgG was weakly correlated with S1-IgG and RBD-IgG. By analyzing the correlation between microneutralization test (MNT) and SARS-CoV-2 specific antibodies detection, result showed that MNT were strongly correlated with ACE2-RBD inhibition assay, S1-IgG, and RBD-IgG. Among them, the strongest correlation was observed between MNT and ACE2-RBD inhibition assay, while the correlation with N-IgG was weak. As is reported that neutralizing antibody of SARS-CoV-2 are all targeted in S protein [10,11,12,13,14,15], S1-IgG, and RBD-IgG in sera could be strong correlated with the result of MNT. However, this magnitude of the difference was significantly greater for the N-specific antibody response compared to all the other relevant antibody responses (S, S1, S2) (*p* < 0.0001), N-IgG in sera could be weakly correlated with the result of MNT [30]. The results of this study further prove it. Based on the above results, ACE2-RBD inhibition assay, S1-IgG and RBD-IgG can all be used as alternative methods for live virus neutralization test to detect the sera neutralizing antibody levels in sera from SARS-CoV-2 vaccinated donors and COVID-19 convalescent patients.

Although many SARS-CoV-2 antibody detection methods have been published, few were a quantitative detection method. In this study, the quantitative detection methods of the ACE2-RBD inhibiting antibody and S1-IgG, RBD-IgG, and N-IgG were established using standards. In this study, both ACE2-RBD binding inhibition percentage rate and ACE2-RBD-inhibiting antibody concentration were used as the evaluation index. When the sera antibody concentration was in the linear range, the results of the two parameters were highly consistent and strongly correlated with each other (Supplementary Figure S2). When the antibody concentration in sera exceeded the linear range, the correlation between the two parameters was weak, and the samples need to be diluted before test. The ACE2-RBD binding inhibition percentage rate detected after sample dilution is not accurate, but the antibody concentration can be calculated. Thus, in this study all sera were 1:20 diluted before detection. The antibody concentration is more accurate as an evaluation index.

In our research, RBD-IgG assay was recommended with higher sensitivity and specificity as a detection method for neutralizing antibodies. Firstly, the SARS-CoV-2 neutralizing monoclonal antibodies are mainly targeted on S1, including the NTD and RBD regions. It has been reported that RBD antibody accounts for 90% of sera neutralizing activity [16], and the NTD antibody is a potential antibody that can cause ADE effect and enhance virus infection [31]. The world’s vaccines mainly come from 13 manufacturers, and the top three vaccines are from Oxford/AstraZeneca, Pfizer/Biontech and Moderna. Pfizer/Biontech and Moderna are mRNA vaccines encoding the S protein, and Oxford/AstraZeneca is an adenovirus vector vaccine encoding the S protein [32]. There are three RBD protein subunit vaccines, namely RBD dimer, Soberana02, and Abdala, which can effectively stimulate the body to produce immune protection. Secondly, ACE2-RBD inhibiting antibodies could not represent all neutralizing antibodies in sera. Neutralizing antibodies binding to RBD include two epitopes, ACE2 competitive epitope and ACE2 noncompetitive epitope. 99% (118/119) of the sera samples from convalescent patients tested in this study had ACE2-RBD inhibiting antibodies, while only (3/26) 11.6% of the samples tested in another report had ACE2-RBD inhibiting antibodies [15]. It is required more sera tests to verify. Therefore, RBD-IgG antibody concentration can more accurately reflect sera neutralizing antibody levels.

One patient in our research showed neutralizing antibody positive with a low serum neutralizing titer of 1:28 in MNT. However, the ACE2-RBD-inhibiting antibody concentration and ACE2-RBD binding inhibition, S1-IgG, RBD-IgG, and N-IgG concentration were under the cutoff value (Figure 2A–E), which were 232 ng/mL, 121 ng/mL, 82 ng/mL, and 263 ng/mL, respectively. In addition, seven samples were detected negative for neutralizing antibody (<1:10), while the detection results of ACE2-RBD inhibiting antibody concentration, ACE2-RBD binding inhibition, S1-IgG concentration, and RBD-IgG concentration were higher than the cutoff value (Figure 2F). ACE2-RBD inhibiting antibody concentration range from 527 ng/mL to 1088 ng/mL. Both the negative and positive coincidence rates between the method and MNT were above 98%, and the kappa coefficient was 0.9586 (*p* < 0.01), which was highly consistent with the neutralization test and could effectively reflect the level of neutralizing antibodies in the sera of COVID-19 convalescent patients and vaccinated donors.

Neutralizing antibody titer in sera is an important indicator for the evaluation of the population immunization effect. It is reported that a 50% protective neutralization level is equivalent to a neutralization titer between 1:10 and 1:30 in sera, which estimates approximately 54 international units (IU)/mL (95% CI 30–96 IU/mL) [33]. Four rapid neutralizing antibody detection methods developed in this study showed the relationship between antibody neutralization titer and neutralizing antibody concentration, and reported the results as ng/mL. However, ng/mL is a physical measure of the quantity and cannot reveal the biological activity of the anti-SARS-CoV-2 antibody. To expand the applicability of these assays, we would calibrate our antibody standard against an international unit system.

In summary, we designed an ACE2-RBD inhibition assay and S1-IgG, RBD-IgG, and N-IgG detection assays based on the chemiluminescence technique to identify potentially neutralizing antibodies in sera. For the first time, a SARS-CoV-2 specific monoclonal antibody was used as a standard to construct the standard curve, which realized the quantitative detection of sera neutralizing antibodies. These four detection methods were evaluated with sera from the SARS-CoV-2 vaccinated donors and COVID-19 convalescent patients. All four detection methods had good sensitivity and specificity. The ACE2-RBD inhibition assay, S1-IgG assay and the RBD-IgG assay had good correlation with the microneutralization test. Thus, we recommend RBD-IgG assay with higher sensitivity and specificity as a detection method for neutralizing antibodies and suggest that it could be used for large scale detection to evaluate the effect of SARS-CoV-2 vaccination.

## Figures and Tables

**Table 1:** Correlation among ACE2-RBD-inhibiting antibody, SARS-CoV-2 specific IgG and microneutralization test results of the sera from 119 convalescent patients.

Spearman Correlation Analysis		Microneutralization Test	ACE2-RBD Inhibiting Assay		Specific IgG Detection		
		IC50 Titer	Antibody Concentration	Inhibition	S1-IgG Concentration	RBD-IgG Concentration	N-IgG Concentration
Microneutralization Test	IC50 Titer		0.8785	0.8792	0.8395	0.8391	0.4276
ACE2-RBD Inhibiting Assay	Antibody Concentration	0.8785		0.9992	0.8699	0.8934	0.4159
	Inhibition	0.8792	0.9992		0.8697	0.8927	0.4152
Specific IgG Detection	S1-IgG Concentration	0.8395	0.8699	0.8697		0.9549	0.3643
	RBD-IgG Concentration	0.8391	0.8934	0.8927	0.9549		0.3750
	N-IgG Concentration	0.4276	0.4159	0.4152	0.3643	0.3750	

**Table 2:** Correlation among ACE2-RBD inhibiting antibody, SARS-CoV-2 specific IgG and microneutralization test results of the sera from 86 vaccinated donors.

Spearman Correlation Analysis		Microneutralization Test	ACE2-RBD Inhibiting Assay		Specific IgG Detection		
		IC50 Titer	Antibody Concentration	Inhibition	S1-IgG Concentration	RBD-IgG Concentration	N-IgG Concentration
Microneutralization Test	IC50 Titer		0.7790	0.7792	0.7408	0.7322	0.4863
ACE2-RBD Inhibiting Assay	Antibody Concentration	0.779		1.0000	0.9411	0.9414	0.4674
	Inhibition	0.7792	1.0000		0.9412	0.9413	0.4674
Specific IgG Detection	S1-IgG Concentration	0.7408	0.9411	0.9412		0.9833	0.4952
	RBD-IgG Concentration	0.7322	0.9414	0.9413	0.9833		0.4776
	N-IgG Concentration	0.4863	0.4674	0.4674	0.4952	0.4776	

**Table 3:** The consistency analysis of chemiluminescence Immunoassays and microneutralization rest results.

	ACE2-RBD Inhibiting Conc.	Inhibition	S1-IgG Conc.	RBD-IgG Conc.	N-IgG Conc.
Specificity	95.75%	95.75%	96.08%	97.06%	95.10%
Sensitivity	99.49%	99.49%	99.49%	99.49%	100.00%
Positive coincidence rate	99.49%	99.49%	99.49%	99.49%	100.00%
Negative coincidence rate	95.75%	95.75%	96.08%	97.06%	95.10%
Total coincidence rate	97.22%	97.22%	97.42%	98.02%	97.02%
P(observed)	0.9722	0.9722	0.9742	0.9802	0.9702
P(chance)	0.5215	0.5215	0.5215	0.5215	0.5215
Kappa Value	0.9419	0.9419	0.9461	0.9585	0.9378

## Data Availability

Data may be made by available through contact with the corresponding author.

## Supplementary Materials

The following are available online at https://www.mdpi.com/article/10.3390/v13081508/s1: Figure S1, The analysis of a receiver operating characteristic (ROC) curve based on positive divided by negative (P/N) values; Figure S2, Correlation of ACE2-RBD binding Inhibition and ACE2-RBD-Inhibiting antibody concentration; Table S1, The level of neutralization antibody in randomly collected sera from COVID-19 convalescent patients or vaccinated donors; Table S2, The analysis of a receiver operating characteristic (ROC) curve based on positive divided by negative (P/N) values; Table S3, Comparison of Chemiluminescence Immunoassays and Microneutralization Test Results.
