# Supplementary material for: Chemiluminescence Immunoassay Based Serological Immunoassays for Detection of SARS-CoV-2 Neutralizing Antibodies in COVID-19 Convalescent Patients and Vaccinated Population
Source: Viruses. 2021 Jul 30;13(8):1508. doi: 10.3390/v13081508 (PMC8402865; doi:10.3390/v13081508)
Supplement: Supplementary file 1 [file viruses-13-01508-s001.zip › viruses-1277546-supplementary.pdf]

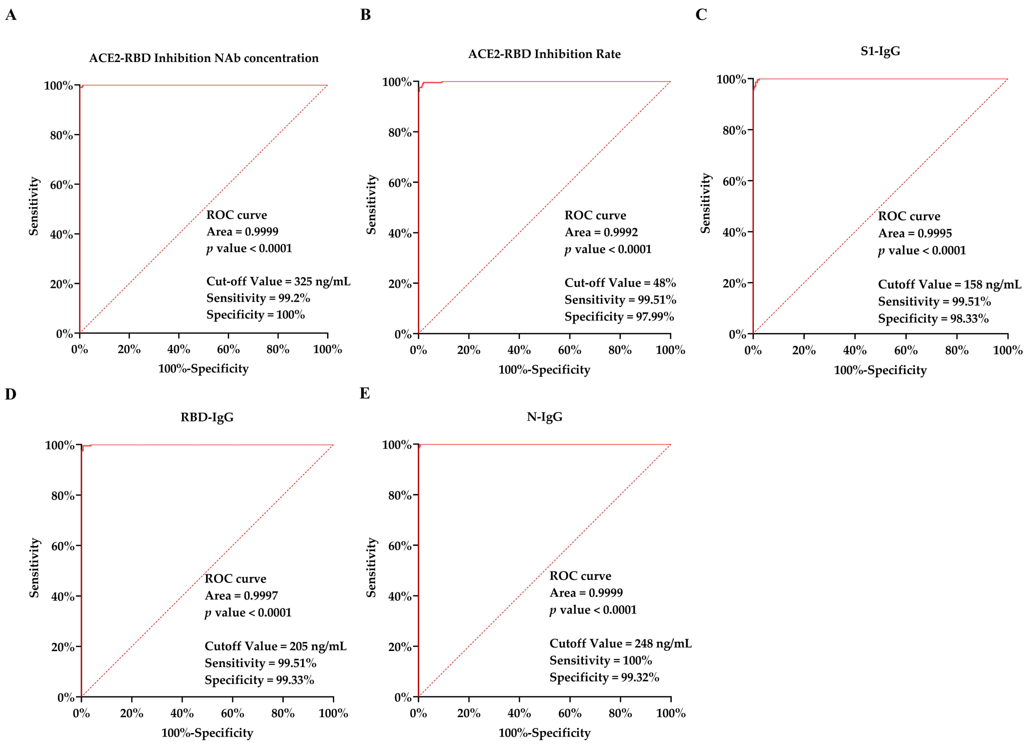

Figure S1. The analysis of a receiver operating characteristic (ROC) curve based on positive divided by negative (P/N) values

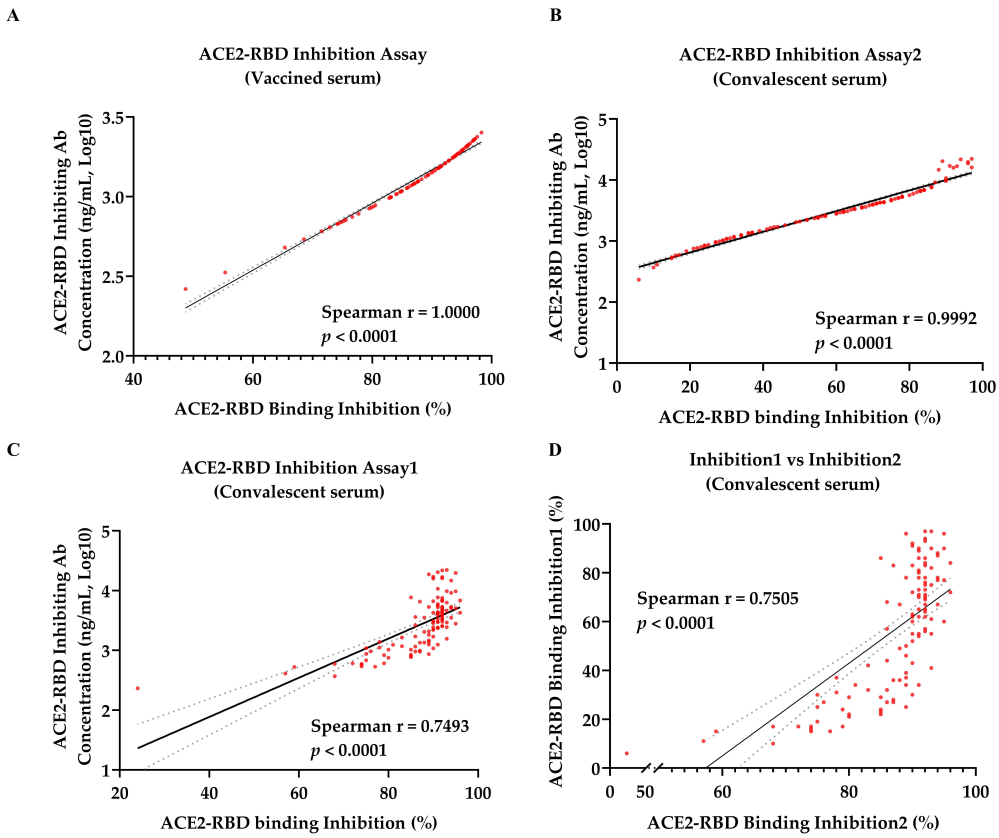

Figure S2. Correlation of ACE2-RBD binding Inhibition and ACE2-RBD-Inhibiting antibody concentration. **(A)** Correlation of ACE2-RBD binding Inhibition and ACE2-RBD-Inhibiting antibody concentration of 86 vaccined samples before dilution; **(B)** Correlation of ACE2-RBD binding Inhibition and ACE2-RBD-Inhibiting antibody concentration of 119 convalescent samples after dilution; **(C)** Correlation of ACE2-RBD binding inhibition and ACE2-RBD-inhibiting antibody concentration of 119 convalescent samples before dilution; **(D)** Correlation of ACE2-RBD binding inhibition before dilution and ACE2-RBD binding inhibition after dilution of 119 convalescent samples.

Table S1. The level of neutralization antibody in randomly collected sera from COVID-19 convalescent patients or vaccinated donors

Neutralization antibody titer	COVID-19 convalescent patients	Vaccinated population
＜1:10	5.88% (7/119)	0%（0/86）
1:10-1:160	42.86% (51/119)	58.14%（50/86）
1:160-1:500	32.77% (39/119)	30.23%(26/86)
＞1:500	18.49%(22/119)	11.63%(10/86)

Table S2. The analysis of a receiver operating characteristic (ROC) curve based on positive divided by negative (P/N) values

ROC curve		Cutoff value	Sensitivity% (95% CI)	Specificity%(95% CI)
ACE2 -RBD Inhibition Assay	Antibody concentration	325ng/mL	99.02 (96.51-99.83)	100(98.73-100.0)
	Inhibition	48.00%	99.51(97.29-99.97)	97.99(95.69-99.08)
Specific IgG Detection	S1-IgG concentration	158ng/mL	99.51(97.29-99.97)	98.33(96.15-99.28)
	RBD-IgG concentration	205ng/mL	99.51(97.29-99.97)	99.33(97.59-99.88)
	N-IgG concentration	248ng/mL	100(98.16-100.0)	99.32(97.54-99.88)

Table S3. Comparison of Chemiluminescence Immunoassays and Microneutralization Test Results

		MNT		Sum
		+	-	
ACE2-RBD binding Inhibition Assay	+	197(A)	13(B)	209
	-	1(C)	293(D)	301
Sum		198	306	504

		MNT		Sum
		+	-	
ACE2-RBD Inhibiting antibody Assay	+	196(A)	13(B)	210
	-	2(C)	293(D)	294
Sum		198	306	504

		MNT		Sum
		+	-	
S1-IgG Detection Method	+	197(A)	12(B)	209
	-	1(C)	294(D)	295
Sum		198	306	504

		MNT		Sum
		+	-	
RBD-IgG Detection Method	+	197(A)	9(B)	206
	-	1(C)	297(D)	298
Sum		198	306	504

		MNT		Sum
		+	-	
N-IgG Detection Method	+	198(A)	15(B)	213
	-	0(C)	291(D)	291
Sum		198	306	504

*Sensitivity=A/(A+C)×100%, Specifity=D/(B+D)×100%.
